# Biomechanical features of drop vertical jump are different among various sporting activities

**DOI:** 10.1186/s12891-022-05290-0

**Published:** 2022-04-08

**Authors:** Kengo Harato, Yutaro Morishige, Shu Kobayashi, Yasuo Niki, Takeo Nagura

**Affiliations:** 1 Department of Orthopedic Surgery, Keio University School of Medicine, 35 Shinanomachi, Shinjuku-ku, Tokyo, 160-8582 Japan; 2 Department of Clinical Biomechanics, Keio University School of Medicine, 35 Shinanomachi, Shinjuku-ku, Tokyo, 160-8582 Japan

**Keywords:** Drop vertical jump, Sport-specific movement, Anterior cruciate ligament, Female athletes, Biomechanics

## Abstract

**Background:**

Risk for non-contact anterior cruciate ligament (ACL) injury can be assessed based on drop vertical jump (DVJ). However, biomechanics of DVJ may differ with various sporting activities. The purpose of the present study was to clarify whether biomechanical features of DVJ are different among various sporting activities in female athletes.

**Methods:**

A total of 42 female athletes, including 25 basketball, 8 soccer and 9 volleyball players, participated in the current investigation. DVJ was done for each female athlete using a three-dimensional motion analysis system which consisted of six cameras, two force plates and 46 retro-reflective markers. Kinematic and kinetic data were recorded for both limbs in each athlete. Simultaneously, frontal and sagittal plane views of the DVJ were recorded using two different high-resolution video cameras to evaluate Landing Error Scoring System (LESS) score. Three-dimensional biomechanical parameters at the knee joint and LESS were compared among three different sporting activities using ANOVA or Kruskal–Wallis test after confirming normality assumption. Thereafter post hoc Tukey or Steel–Dwass was utilized for multiple comparison.

**Results:**

Soccer players had better LESS score, and peak knee flexion angle was significantly larger in soccer players compared to the other sports. In addition, knee abduction angle at initial contact (IC), peak knee abduction angle, knee internal rotation angle, and knee abduction moment within 40 ms from IC were significantly smaller in soccer players, compared to basketball players. In terms of volleyball players, knee abduction angle at IC and knee internal rotation angle at IC were significantly larger than soccer players, whereas no significant biomechanical differences were found between basketball and volleyball players.

**Conclusions:**

From the present study, female basketball and volleyball players have worse LESS score, smaller peak knee flexion angle, greater knee abduction angle at IC and greater knee internal rotation angle at IC, compared to female soccer players. Thus, female basketball and volleyball players may have an increased risk of non-contact ACL injury during the jump-landing task, compared to soccer players. Biomechanics of DVJ depends on characteristics of the athlete's primary sport.

## Background

Non-contact anterior cruciate ligament (ACL) injuries are common especially for young female athletes. Clinically, non-contact ACL injuries include deceleration, lateral pivoting, or landing tasks which are associated with high external knee joint loads. According to previous literature, drop vertical jump (DVJ) can be a useful screening tool to evaluate the risk for non-contact ACL injury in female athletes [1]. For example, Hewett et al. suggested that female athletes with larger knee abduction angle at initial contact (IC), peak knee abduction angle, and peak knee abduction moment during DVJ would be prone to non-contact ACL injury [1]. The DVJ has thus been used to assess the risk for non-contact ACL injury in many subsequent studies [2–17]. On the other hand, Krosshaug et al. indicated that DVJ could be a poor screening tool for non-contact ACL injuries from a prospective cohort study [18]. So far, the reason of this difference has been unknown. The participant in Hewett’s study were 205 female adolescent soccer, basketball, and volleyball players [1], whereas those in Krosshaug’s study were elite 372 handball players and 338 soccer players [18]. Therefore, primary sporting activities were different between these studies. Although it is controversial as to whether DVJ is an important screening tool for non-contact ACL injury, little attention has been paid to the biomechanical differences of DVJ among various sporting activities.

The purpose of the current study was to assess whether the characteristics of different sporting activities would affect the biomechanics of DVJ in female athletes. It was hypothesized that biomechanical features would depend on the athlete's primary sport.

## Methods

### Participants

A sample of convenience of 42 female collegiate athletes were enrolled in the present study. They were comprised of twenty-five basketball players, eight soccer players and nine volleyball players. Tegner activity scales were 7 to 9. All of subjects were members of college sports team and their practice was longer than 18 h a week. None of the athletes had any history of surgeries to the trunk and lower extremities based on musculoskeletal problems such as fracture, ligament rupture, and cartilage damage. Female athletes are known to have greater risk of non-contact ACL injury than male athletes [19, 20], and thus, females were chosen in the present study. A written informed consent form approved by Institutional Review Board of our university was obtained from each athlete.

### Test procedures

The jump-landing biomechanics during Drop Vertical Jump (DVJ) was examined. Participants were instructed to stand on top of the box with their feet shoulder width apart. Thereafter, they were instructed to drop off the box from a 30 cm high box forward to a distance of 50% of their height away from the box, land with one foot on each force plate, and then immediately upon landing jump as high as possible. Before data collection, instructions were given on how to perform DVJ. After performing DVJ several times as warm-ups, three successful trials were recorded for each athlete. DVJ was captured using a three-dimensional motion analysis system consisted of six cameras (120 frames/s; Oqus, Qualisys, Sweden) and two force plates (frequency 600 Hz; AM6110, Bertec, Columbus, OH, USA). The force plate collected ground reaction force (GRF) data at 600 Hz and were synchronized to the camera sampling rate of 120 Hz. The time at initial contact (IC) and toe-off (TO) from the jump was identified based on GRF data.

A total of 46 retro-reflective markers (14 mm in diameter) were placed on anatomic landmarks and specific locations. A set of anatomical landmarks were defined as follows; spinous process of vertebra at the level of C 7 and Th 10, jugular notch and xiphoid process of sternum, acromion, anterior superior and posterior superior iliac spine, greater trochanter, medial and lateral femoral epicondyles, medial and lateral malleoli, head of first and fifth metatarsal bone, scaphoid, and calcaneus. Additional specific markers were placed on the frontal and lateral aspects of thigh (4 markers) and shank (4 markers) [21, 22]. Marker trajectories were used to calculate joint centers and segment positions in standard quiet stance, and to track segment motion during the DVJ tests. Joint angles were calculated based on the cardan sequence of XYZ, equivalent to the joint coordinate system.

**Fig. 1:**
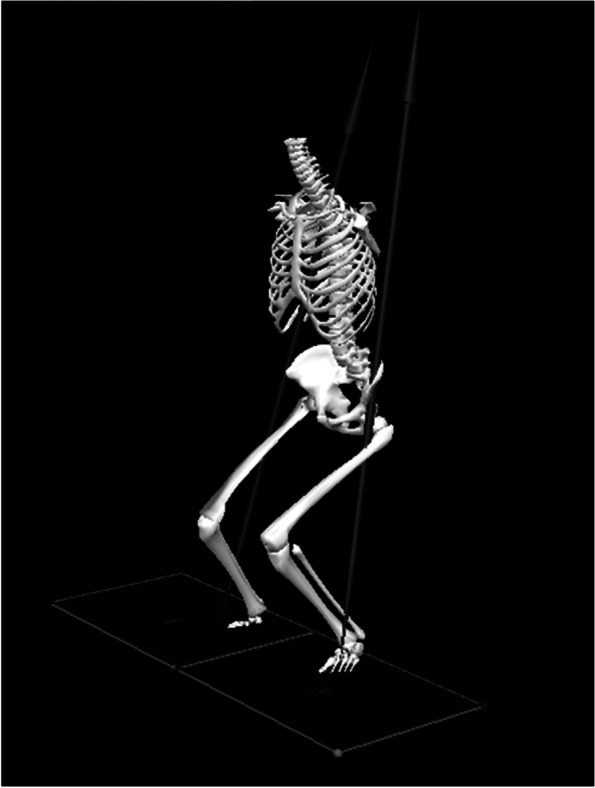
Three-dimensional knee kinematics and kinetics were calculated during initial contact (IC) to Toe-off (TO) in DVJ

For each athlete, three-dimensional kinematic, kinetic and GRF data were assessed bilaterally during IC to TO. Marker movements were recorded by Qualisys Track Manager Software (version 2.7). To calculate biomechanical parameters at the knee joint, Visual 3D (C-motion Company, Rockville, MD) was used (Fig. 1). The following kinematic parameters were used: knee flexion angle at IC, peak knee flexion angle (IC-TO), knee abduction angle at IC, peak knee abduction angle (IC-TO), knee internal rotation angle at IC, and peak knee internal rotation angle (IC-TO). Knee internal rotation was defined as tibial rotation with respect to the femur. The following kinetic parameters were used: peak knee flexion, abduction, internal rotation moments within 40 ms from IC based on the previous study [21, 23]. Simultaneously, frontal and sagittal plane views of the DVJ were acquired using high resolution video cameras (120 frames/s; Oqus, Qualisys, Sweden) to evaluate Landing Error Scoring System (LESS) score.

### Statistical analysis

After Shapiro–Wilk test was performed to confirm normality assumption, ANOVA or Kruskal–Wallis test were used to compare biomechanical parameters between groups. Thereafter, post hoc Tukey or Steel–Dwass were used for multiple comparison. The statistical significance level was set at *P* = 0.05. Demographic data of athletes, kinematic and kinetic data were compared among groups. All statistical analyses were done with the Microsoft Excel Statistical Package, version 2015 (Social Survey Research Information, Tokyo).

## Results

Athletes’ demographic data in each group are shown in Table 1. A total of 50 knees in 25 basketball players, 16 knees in 8 soccer players and 18 knees in 9 volleyball players, were analyzed. There were no significant differences among groups except for age. Soccer players were youngest among groups.

**Table 1:**
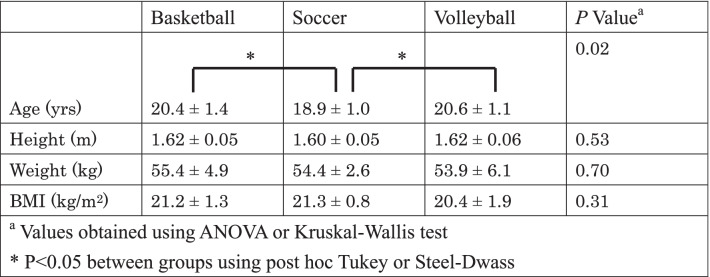
Demographic data of athletes in each group (mean ± SD)

Kinematic and kinetic differences in each group are presented in Tables 2 and 3, respectively. LESS score was 4.8 ± 1.5 in basketball players, 3.3 ± 1.4 in soccer players, and 6.0 ± 2.8 in volleyball players. LESS score was significantly better in soccer players than in volleyball players (*P* = 0.009). In addition, in soccer players, peak knee flexion angle was significantly larger compared to basketball and volleyball players. Therefore, relative stiff landing (less knee flexion) was found in basketball and volleyball players. Moreover, knee abduction angle at IC, peak knee abduction angle, knee internal rotation angle at IC, and peak knee abduction moment within 40 ms from IC were significantly smaller in soccer players than in basketball players. In terms of volleyball players, knee abduction angle at IC and knee internal rotation angle at IC were significantly larger than soccer players, whereas no significant kinematic and kinetic differences were found between basketball and volleyball players.

**Table 2:**
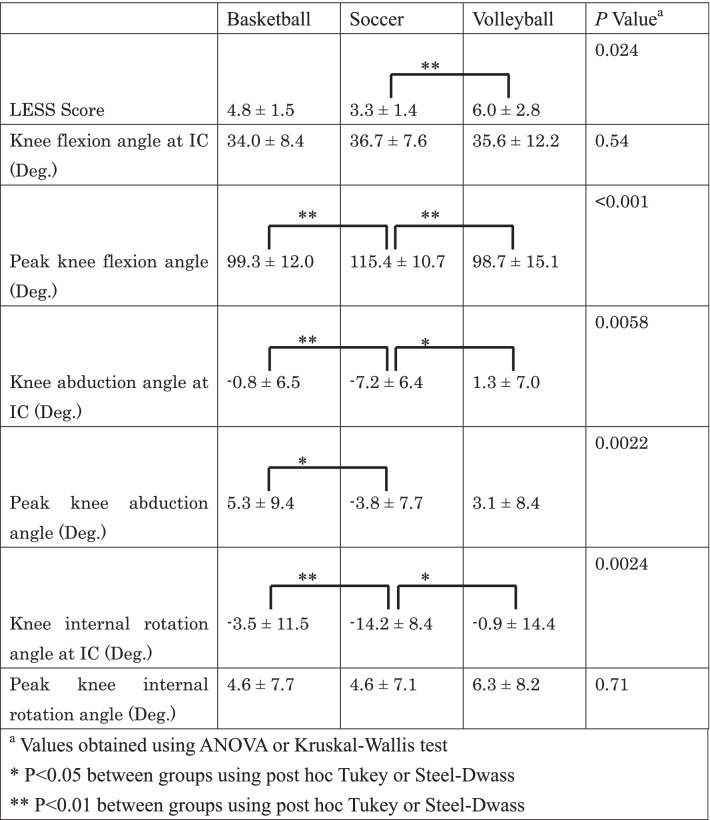
Kinematic differences in each group (mean ± SD)

**Table 3:**
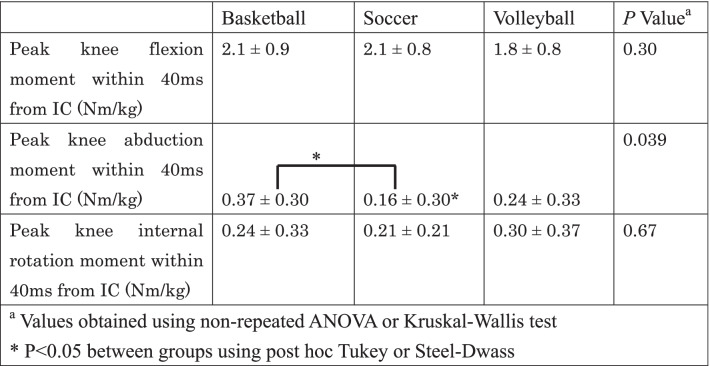
Kinetic differences in each group (mean ± SD)

## Discussion

The results of the present study supported the hypothesis that biomechanical features during DVJ would depend on the athlete's primary sport. The most important finding of the current investigation was that female basketball and volleyball players were likely to perform stiff landing, compared to soccer players.

The LESS score can be evaluated using frontal and sagittal plane views of video data. Therefore, the score has clinically been shown to be a convenient assessment tool of jump-landing biomechanics. Currently, the score during DVJ has been used to evaluate the risk factor for ACL injury of female athletes. Padua et al. suggested that the LESS could be a valid and reliable tool for identifying potentially high-risk movement patterns during a jump-landing task [15], and 5 in LESS score was the optimal cut point, generating a sensitivity of 86% and a specificity of 64% for non-contact ACL injury [14]. In the present study, percentages of collegiate athletes with greater than 5 in the LESS were 64% (16/25) in basketball, 13% (1/8) in soccer, and 67% (6/9) in volleyball players, respectively. Therefore, the potential risk for non-contact ACL injury may be greater in female basketball and volleyball players, compared to soccer players.

Regarding DVJ as a screening test of non-contact ACL injury, controversy remains as described before. For instance, Hewett et al. reported that female athletes (205 female adolescent soccer, basketball, and volleyball players) with increased knee abduction angle or moment during DVJ would be prone to non-contact ACL injury [1]. However, Krosshaug et al. indicated that DVJ could be a poor screening test for ACL injuries in their prospective cohort study using elite 372 handball players and 338 soccer players, as knee abduction angle at IC, peak knee abduction moment, and peak knee flexion were not associated with the increased risk for ACL injury [18]. Although the true reason of this difference between these studies is unclear, absolute value of knee abduction moment during DVJ seemed to be greater in Hewett’s study than in Krosshaug’s study. Therefore, biomechanical features might be different based on participants' primary sport. Braun et al. assessed ACL biomechanical risk factors in female field hockey and lacrosse players to determine whether sport-specific posture might contribute to the increased incidence of ACL injury observed in lacrosse athletes [3]. They concluded that decreased knee flexion angle during landing, consistent with sport-specific playing postures, may contribute to the higher incidence of ACL injury in lacrosse players relative to field hockey. Similarly, from the present study, significant differences of kinematic and kinetic changes were found among three different sporting activities. In particular, less knee flexion was found during landing phase of DVJ in basketball and volleyball players, compared to soccer players.

Several limitations should be noted in the present study. First, the sample size was different for each sport. In particular, the sample sizes for soccer and volleyball were much smaller compared to basketball. Second, the present study was done using college athletes with Tegner activity scale 7 to 9. Thus, it is possible that the biomechanical parameters of high school or recreational athletes may be slightly different. Third, only three different sporting activities were included in the current study. The other kind of sports, like hockey, lacrosse, handball and so on, were still unknown. Lastly, although the risk of non-contact injury to the ACL was evaluated, the actual incidence of ACL injury could not be investigated. Nonetheless, the present results provide important information regarding the sport-specific characteristic of female knee kinematics and kinetics during jumping tasks.

## Conclusion

The findings of this study showed that the biomechanics of DVJ depends on characteristics of the athlete's primary sport. In particular, female basketball and volleyball players are likely to perform a stiffer DVJ landing compared to soccer players, which may increase the risk of non-contact ACL injury in jumping tasks.

## Data Availability

The datasets used and/or analysed during the current study available from the corresponding author on reasonable request.

## Abbreviations

ACL: Anterior cruciate ligament
DVJ: Drop vertical jump
GRF: Ground reaction force
IC: Initial contact
LESS: Landing error scoring system
ms: Milliseconds
PKABDM: Peak knee abduction moment
PKFM: Peak knee flexion moment
PKIRM: Peak knee internal rotation moment
TO: Toe-off
vGRF: Vertical ground reaction force
